# Neuronal Entropy Depends on the Level of Alertness in the Parkinsonian Globus Pallidus *in vivo*

**DOI:** 10.3389/fneur.2014.00096

**Published:** 2014-06-25

**Authors:** Daniela Sabrina Andres, Daniel Cerquetti, Marcelo Merello, Ruedi Stoop

**Affiliations:** ^1^Institute of Neuroinformatics, ETH Zurich and University of Zurich, Zurich, Switzerland; ^2^Movement Disorders Section, Institute for Neurological Research Raul Carrea, Fleni Institute, Buenos Aires, Argentina; ^3^Society in Science, The Branco-Weiss Fellowship, ETH Zurich, Zurich, Switzerland

**Keywords:** basal ganglia, Parkinson’s disease, entropy, alertness, emergent properties, non-linear

## Abstract

A new working hypothesis of Parkinson’s disease (PD) proposes to focus on the central role of entropy increase in the basal ganglia (BG) in movement disorders. The conditions necessary for entropy increase *in vivo* are, however, still not fully described. We recorded the activity of single globus pallidus pars interna neurons during the transition from deep anesthesia to full alertness in relaxed, head-restrained, control, and parkinsonian (6-hydroxydopamine-lesioned group-lesioned) rats. We found that during awakening from anesthesia, the variation of neuronal entropy was significantly higher in the parkinsonian than in the control group. This implies in our view that in PD the entropy of the output neurons of the BG varies dynamically with the input to the network, which is determined by the level of alertness. Therefore, entropy needs to be interpreted as a dynamic, emergent property that characterizes the global state of the BG neuronal network, rather than a static property of parkinsonian neurons themselves. Within the framework of the “entropy hypothesis,” this implies the presence of a pathological feedback loop in the parkinsonian BG, where increasing the network input results in a further increase of neuronal entropy and a worsening of akinesia.

## Introduction

The measurement of neuronal entropy can be used to characterize the behavior of neural systems (1–4). The concept of entropy was developed in the field of thermodynamics, where it is related to the number of possible states of a system, and can be interpreted as a measure of disorder. Applying this idea to the study of neural systems, neuronal entropy can be associated roughly with the level of disorder present in the discharge of single neurons. From this follows that neuronal entropy shows a negative relation with the predictability of the occurrence of single spikes or, in other words, with the amount of information transmitted by a neuron in the form of temporal patterns. A field where this approach has yielded particularly successful results is Parkinson’s disease (PD). During the last years, a growing body of evidence has shown that entropy is increased throughout the parkinsonian basal ganglia (BG) (5–7). Furthermore, a correlation between a decrease in neuronal entropy and anti-parkinsonian therapy was found. In MPTP-treated monkeys, the level of entropy of the internal as well as the external globus pallidum (GPi and GPe, respectively) and the motor thalamus (Th) can be reduced by the application of high frequency deep brain stimulation (DBS) (8). In human patients with PD, an inverse relation between neuronal entropy and the effect of apomorphine has also been reported (9). These results indicate that the clinical effectiveness of anti-parkinsonian treatment is correlated with a decrease in neuronal entropy throughout the BG–Th circuit. In this context, it is clear that the evaluation of neuronal entropy is becoming increasingly relevant for the understanding of the pathophysiology of the BG, and might be a key concept in the future for the development of clinical interventions.

A model of BG dysfunctions was recently proposed, which is based on the central role that entropy might play altering the “legibility” of information transmitted through the BG (10). Developing the so called “entropy hypothesis,” the authors suggest that the progressive motor impairment in PD might be related to an increasing “illegibility” of neuronal information. The entropy hypothesis is mainly based on two facts: first, anti-parkinsonian treatments decrease the abnormally high entropy in the parkinsonian BG, and second, hyperkinetic conditions such as dystonia are associated with an abnormally low entropy level in the BG (8, 9, 11). Therefore, hyper- and hypo-kinetic movement disorders seem to be placed at the opposite extremes of a spectrum characterized by the level of entropy of the neuronal discharge of the BG. However, up to now the conditions necessary for the emergence of this phenomenon have not been described. To better understand the changes underlying the increased entropy of the BG in PD, we analyze the variation in the level of entropy in the neuronal discharge of the rat GPi during the transition from deep anesthesia to relaxed, full alertness. Since GPi neurons are essentially output nodes of the BG network, any modification of the information/entropy content in their discharge will be reflected in a necessary disruption of the communication between the BG and the Th/cortex. By studying the GPi during the awakening from anesthesia, we may gain insight into the way that the BG respond to an increasing level of un-specific input *in vivo* in the healthy vs. the parkinsonian condition.

Our study is motivated by the application of the surgical treatment of PD, and in particular DBS, a therapy that has been successful in alleviating PD symptoms in selected groups of patients for decades (12–14). The implantation surgery of DBS electrodes takes place usually while the patient is awake, under local anesthesia only (15, 16). Under these conditions, microrecording of the neuronal activity along the surgical tract is the gold standard procedure for the identification of the surgical target (17–19). In previous works, we have emphasized the importance of a careful characterization of signals obtained from the patients’ own brains, for both research and clinical purposes (20, 21). However, the fact that the subjects are alert during the recording makes the interpretation of these data difficult, since measured properties can be influenced by the alertness state as well as the severity of the disease. In the human BG in patients with PD, contradictory results have been reported between alertness and deep anesthesia (22–25). In animals, on the other hand, the discrimination between the effect of the level of alertness and that of sensory stimuli or motor activity remains technically challenging (26–28). Our experiments were designed to mimic the human surgery in PD patients as much as possible. With the aim of exploring the effect of alertness over the healthy and parkinsonian GPi, all the neuronal recordings were obtained with the animals relaxed, under environmental silence and with their eyes covered, guaranteeing the absence of motor activity, as well as any auditory and visual stimuli at the times of the experiments.

## Materials and Methods

### Ethics statement

All animal experiments and procedures were conducted with adherence to the norms of the Basel Declaration (29). The experimental protocol was revised and approved by our local ethics committee CEIB, Buenos Aires, Argentina. All experiments took place at the authorized laboratories of our center, and adherence to the Basel Declaration standards were monitored by our research staff. During the time previous and between experiments, animals were housed in racks with optimal temperature, pressure, and air humidity regulation under an inverted 12 h light cycle, with water and food available *ad libitum*. To minimize animal suffering, optimal anesthetic and analgesic medication were used as described below. Euthanasia was conducted using a high dose of meperidine, an opioid suitable for that purpose, guaranteeing the absence of animal suffering during the procedure. In compliance with the 3-R principles, the number of animals used in the experiments was the minimum considered necessary to achieve sound conclusions.

### Animal model

Adult male and female Sprague-Dawley rats weighing 250–350 g were randomly divided in three groups: 6-hydroxydopamine-lesioned group (6OHDA), Sham-lesioned group, and not-lesioned group. The choice of the three experimental groups obeyed the following rationale: the 6OHDA-lesioned group modeled the parkinsonian case and the healthy group served as a general control group, while the comparison of these two groups with the Sham-lesioned group guaranteed that the behavioral deficits in the parkinsonian model were due to the chemical action of the toxin and not to any other effect product of the surgical procedure itself. Animals within the 6OHDA group were lesioned unilaterally following the partial-lesion model originally described by Sauer and Oertel (30). A total dose of 20 μg of 6OHDA diluted in 4 μl of saline solution supplemented with 0.2 mg/ml of ascorbic acid was injected at a rate of 1 μl/min in the striatum (Str – coordinates: anterio-posterior, +1.0; lateral, +3.0; depth, −4.5) using a Hamilton microsyringe. After waiting 5 min from the time, the injection was completed, the syringe was withdrawn from the lesioning site at a rate of 1 mm/min. Animals in the Sham-group underwent the same surgical procedures as the 6OHDA group, but were injected only with the vehicle (ascorbic acid solution, at the same concentration mentioned above). All 6OHDA and Sham lesions were placed in the left hemisphere. During the surgery, temperature maintenance was achieved with the use of electrical pads. The surgery was conducted with the aid of a stereotactic frame (Small Animal Stereotaxic Instrument, LS900, David Kopf Instruments, Tujunga, CA, USA) and coordinates were assessed by use of the Paxinos and Watson Atlas (31). Animals were placed in the frame and reference points were defined. In the horizontal plane, the skull point bregma was taken as the reference point. Its position was assessed with the help of an optical microscope. The cortical surface was considered the reference point along the vertical axis, and its position was defined with the aid of electrical means.

Between 21 and 28 days after the lesion procedure, animals were evaluated using the cylinder test (32), which served the purpose of quantifying the asymmetry in motor behavior. The animals were placed in a transparent cylinder for 5 min and left to explore freely, and only weight supporting touches of the wall were counted, according to the criteria described by Lundblad et al. (33) for the cylinder test. All tests were video-recorded. Animals were killed after the recording-surgery, using a high dose of meperidine, an opioid of choice for euthanasia procedures.

Brains were post-fixed in paraformaldehyde 4% for 24 h, then dehydrated in ethanol 60% and embedded in paraffin. Sections with a thickness of 3 μm were obtained with a microtome (Leica RM 2235) on Superfrost plus slides, sections were dried over night at 37°C. Immunohistochemical staining of the substantia nigra pars compacta was performed using tyrosine hydoxylase antibody to investigate dopamine loss (polyconal anti-rabbit, Millipore IHCR1003-6). The sections were de-paraffinized and heat antigen retrieval was performed with citrate buffer pH 6.0 (Dako, Target Retrieval Solution pH 6.0) for 15 min in a microwave. Sections were rinsed in PBS and briefly stained with Gill’s Hematoxylin, rinsed with water, endogenous peroxidase activity was quenched for 10 min in 3% H_2_O_2_ at room temperature. After rinsing with PBS, blocking was performed with Protein-Block (Dako Protein-Block serum free) for 10 min. Primary antibody incubation (non-diluted according to datasheet) was done at room temperature for 60 min, followed by three times wash with PBS. Dako Dual Link (Dako EnVision System HRP, Anti-Mouse/Rabbit Dual Link System) was used as a secondary antibody and incubated for 30 min at room temperature, followed by a three times PBS wash. Tyrosine-hydroxylase was revealed staining with AEC-Kit HRP Substrate (Invitrogen, ready to use) for 15 min, followed by a PBS rinse. Covers lips were mounted with Kaiser’s Glycerin Gelatine (Merck Chemicals).

### Anesthesia, analgesia, and antibiotic medication

Three complementary drugs were used for achieving anesthesia and analgesia in our study: chloral hydrate, tramadol, and lidocaine. Animals were injected with a 300-mg/kg intraperitoneal dose of chloral hydrate (at a concentration of 50 mg/ml) used as anesthetic. At this dose and concentration of chloral hydrate, a mortality rate of 0% in adult rats has been reported, while sufficiently deep anesthesia for surgical procedures is achieved (34). Anesthesia can be defined as the concomitant presence of unconsciousness, analgesia, and muscle relaxation (35). In current approaches to anesthesia, these effects are usually obtained with combinations of multiple drugs, since this allows using lower doses and therefore minimizing morbimortality (36). Chloral hydrate is a well-known sedative with potent hypnotic effects, widely used not only in veterinary medicine but also in pediatric and neonatal medicine (37–39). However, it does not have important analgesic effects. In the current protocol, analgesia was achieved with a 4-mg/kg dose of intraperitoneal tramadol. Tramadol is a drug commonly used in veterinary medicine, which has combined mechanisms of action, a wide safety rank, few side effects and has proven effective for managing surgical pain (40–43). Tramadol has been shown to have an analgesic potency similar to meperidine and morphine for treating pain of different origins, including surgical pain (44, 45). In our protocol, the tramadol dose was repeated between 12 and 24 h after the surgery to maintain analgesia, and therefore, it was used both as pre-emptive analgesic and as post-operative medication. Local anesthesia (lidocaine) was used at the incision and at contact points. The eyes of the animals were protected from corneal drying with ophthalmic solution drops. Antibiotic prophylaxis was administered in the form of a single 10 mg/kg dose of intramuscular cefazoline previous to the surgery.

The choice of the mentioned drug profile responded to particular issues related to the disease model implemented (6OHDA-lesion model of PD). Other drug options in laboratory animals include dissociative anesthetics (in particular ketamine and combinations of this drug, for example ketamine–xylazine), barbiturates, and inhalant anesthetics (halothane, isoflurane, sevoflurane, among others). Ketamine–xylazine has been reported to yield neuroprotective effects over the central nervous system (CNS), and it is currently questioned to what degree it interferes with the 6OHDA PD model, which made it unsuitable for the present study (46). In the case of isoflurane, it has been shown to induce apoptosis in the CNS (47, 48), which could also have potentially affected the implementation of the 6OHDA-model. Since other inhalant anesthetics have not been analyzed regarding this effect, it is safer to avoid this drug family. Halothane might have been an alternative, but it is a highly hypotensive and arrhythmogenic drug, not necessarily safe in small animals (49).

The anesthetic and analgesic medication used was the same for the lesion- and the neuronal activity recording surgeries, and the stereotactic procedure was repeated as well.

### Assessment of anesthesia depth

During the neuronal activity recording surgery, the state of consciousness was characterized periodically (every 10–12 min) by evaluating the tail-pinch reflex with the application of a standardized non-painful stimulus. Methods to assess the state of consciousness under anesthesia in animals have been widely discussed (50, 51). We defined the following levels of alertness. Level 1: deep anesthesia, level 2: mild alertness, level 3: full alertness. At level 1, animals did not present a positive paw withdrawal reflex, cutaneous reflex, or tail-pinch reflex. Level 2 was defined as the first time of appearance of a positive tail-pinch reflex, and at level 3 the animals showed a strong response to tail-pinch stimulation, either withdrawing both paws and/or energetically contracting abdominal muscles. Since the evaluation of reflexes is subjective, all the evaluations were conducted by the same person, to avoid inter-personal variation. At the end of the recording surgery, we confirmed the wellness and alertness level of the animals by letting them explore freely through the laboratory.

### Recording of neuronal activity

After completed motor evaluation, animals went through stereotactic surgery with the objective of registering spontaneous activity of the medial globus pallidus or globus pallidus pars interna (mGP or GPi). This nucleus corresponds to the structure that was previously called entopeduncular nucleus. In the present work, we adhere to the nomenclature proposed by the latest edition of Paxinos and Watson’s Atlas and refer to this structure as mGP or GPi. The time between the lesion and the recording surgery was between 21 and 28 days. This amount of time has been proven to account for a significant level of dopamine-cells and dopamine-content loss in comparison to the contralateral, not-lesioned side of the brain (30). Similar times were allowed between both surgeries for the Sham-group. Following anesthesia, animals were placed in a specially designed restraining device, which was built *ad hoc* with semi-rigid plastic and covered in the inside with a soft and high quality thermal insulator. The device’s purpose was not to keep the animals firmly restrained if they spontaneously moved, as it was only loosely bound. On the contrary, it served the purpose of minimizing discomfort helping animals to stay calm during the surgery. During the whole recording time, the animals did not make any spontaneous movements. If the animals did not relax but, on the contrary, attempted to move during the experiment, we considered that an end-point for the recording-surgery. During the whole surgical procedure, the eyes of the animals were covered and all surgeries were conducted in identical conditions of environmental silence. Following these procedures, we were able to record neuronal activity uninterruptedly during the awakening process and for long periods of time, obtaining up to 3 h of recording of the same neuron. Recording coordinates fell within the limits defined as the GPi by Paxinos and Watson’s Atlas. Neuronal recordings were obtained using glass-insulated platinum/iridium (Pt/Ir 80/20%) microelectrodes with nominal impedance of 0.8–1.2 MΩ (mTSPBN-LX1, FHC, Inc., Bowdoin, ME, USA). Signals were amplified, conditioned and monitored with an analog oscilloscope, digitized with a dedicated acquisition system (1401plus, CED) and saved in a PC running Spike 2.0 software. The sampling rate was 20 kHz and total amplification including probe was ×10,000, checked with a built-in calibration signal of 1 mV p–p at the beginning of each experiment.

### Data analysis

Signals were processed off-line. Spikes were extracted and classified using the algorithm developed by Quian Quiroga (52). Single units were used to construct interspike intervals (ISI) time series. In order to guarantee stationary conditions, 30 s of recording following the application of a tail-stimulus were discarded from each time series. To be able to characterize different alertness levels unaffected by the proper transition between them, the time separation between the recording segments analyzed was maximized. For alertness levels 1 and 3, the first and the last segments possible at the beginning and at the end of the recording were selected, respectively. In this way, a time separation of 22.52 ± 3.79 min (mean ± SEM) was obtained between successive recording segments. Although the transition segments were not used for the analysis, the recordings were uninterrupted during the whole awakening process with the objective of guaranteeing that the same neuron was being recorded at different alertness levels.

We quantified the bursting activity by counting the percentage of spikes that triggered a burst (Burst Triggering Spikes, BTS). We applied the rank-surprise algorithm to achieve burst detection (53). Based on this algorithm, we defined the BTS-index as the percentage of bursts over the total number of spikes. As a value limit for the largest ISI to be considered to be part of a burst, we used the p75 of the ISIs distribution. The minimum surprise value accepted was α = −log(0.01). Entropy was measured using the sample entropy (SE) algorithm developed by Richman and Moorman (54). We analyzed the dependence of the SE with the length of the recording (number of ISIs, *n*) and the embedding dimension (*m*). We chose a tolerance parameter equal to 0.15 SD (SD, standard deviation of the ISIS distribution). The percentage entropy variation for a given alertness transition was calculated as:

(1) $$\Delta \text{SE}=\frac{\text{S}{\text{E}}_{\text{2}}-\text{S}{\text{E}}_{\text{1}}}{\text{S}{\text{E}}_{\text{1}}}\;\;\times \;\;100,$$

where SE_2_ is the SE at a given alertness level and SE_1_ is the SE at the previous one. Statistical comparisons between groups were performed using the two-sample *t*-test, and differences were considered significant for *p* ≤ 0.05.

## Results

### Animal model

**Figure 1:**
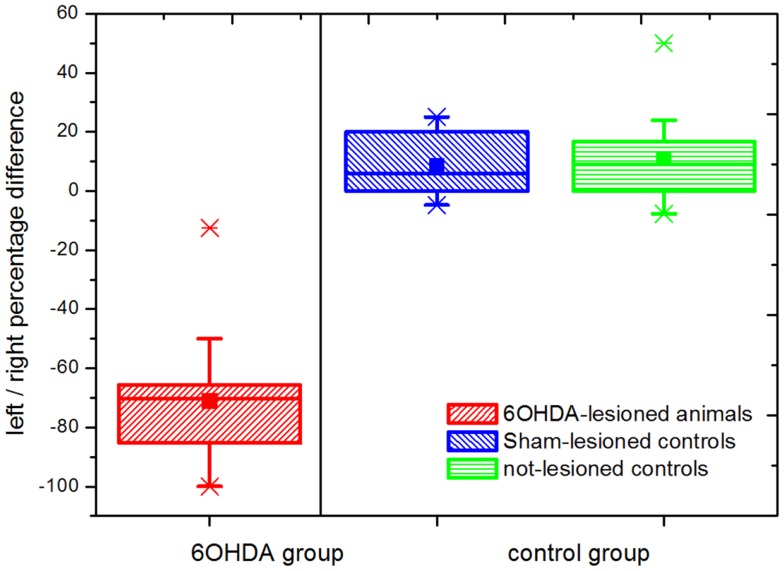
**Cylinder test results**. Percentage differences in weight supporting wall touches with the left (negative) vs. right (positive) paw are shown. Statistically significant differences were observed between the parkinsonian model (*n* = 16) and both the Sham-lesioned (*n* = 10) and the not-lesioned (*n* = 10) control groups (*p* ≤ 0.05). This not-overlapping behavioral differences between parkinsonian (6OHDA-lesioned) animals and both control groups follow the correct implementation of the model. The boxes edges represent percentile 75 (p75) and percentile 25 (p25), respectively, the inner line indicates the median and whiskers account for p75 + 1.5 (p75–p25) and p25–p1.5 (p75–p25) respectively. The mean is represented by the inner, solid box. Crosses indicate outliers.

Figure 1 shows the results of the behavioral evaluation through the implementation of the cylinder test for the three animal groups analyzed. We registered a total of 22.17 ± 1.68 (mean ± SEM) touches/test. The left (negative) to right (positive) bias was calculated as the percentage difference between left and right touches over total number of touches. Animals in the 6OHDA group (parkinsonian model) showed a marked asymmetry toward the side ipsilateral to the lesion, reflected by a left bias in the use of the front-limb for weight supporting touches of the wall. On the contrary, both control groups (the Sham- and the not-lesioned groups) showed a right bias. A statistical difference with a *p*-value <0.01 was obtained between the 6OHDA-lesioned and both the Sham- and the not-lesioned groups, while no significant difference was observed between the last two. The absence of overlap seen between the 6OHDA-lesioned and both control groups evidenced the successful implementation of the 6OHDA- vs. Sham-lesion model.

### Neuronal recordings

**Figure 2:**
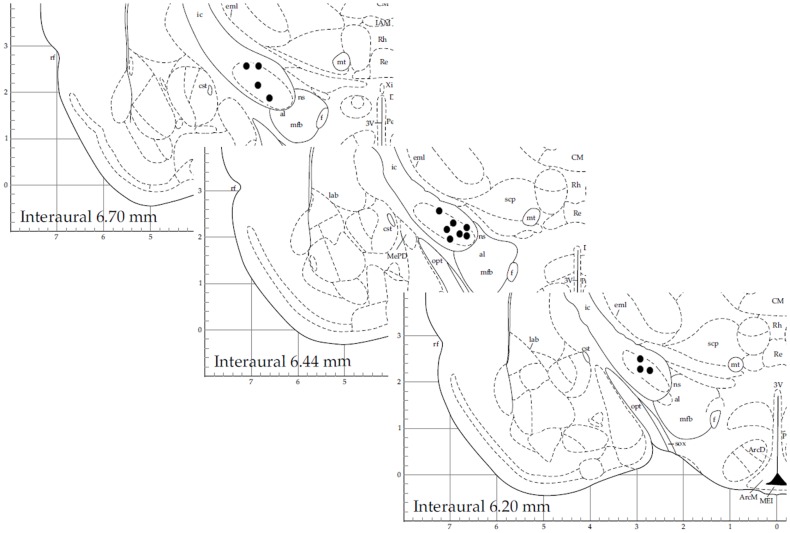
**Stereotactic coordinates of recording sites for 14 sample neurons**. This figure is modified from Ref. (31) with permission.

**Figure 3:**
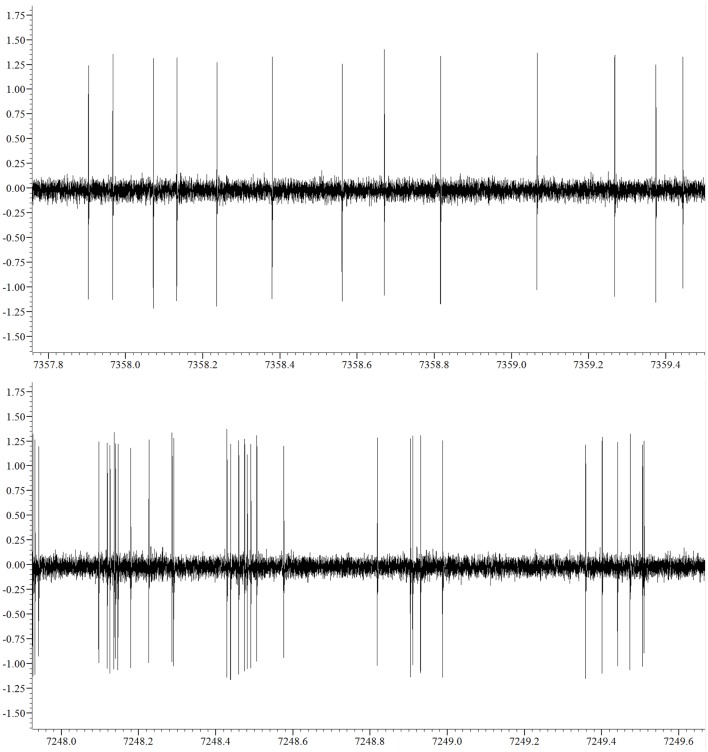
**Sample non-bursting (upper panel) and bursting (lower panel) segments of the neuronal activity recorded**.

We obtained a total of 38 recordings, 17 obtained from 16 6OHDA-lesioned animals and 21 from 20 control animals (10, Sham and 10, not-lesioned). Eight recordings covered the 3 studied levels of alertness, 13 covered the transition from alertness level 1–2, and 17 covered the transition from alertness level 2–3. For our analysis, 21 recording segments obtained under deep anesthesia (11 controls, 10 parkinsonian), 37 under mild alertness (17 controls, 20 parkinsonian), and 24 at full alertness (13 controls, 11 parkinsonian) were included. Recording sites can be seen in Figure 2, as determined by stereotactic coordinates. Figure 3 shows sample recording segments of bursting and tonic-firing activity. The mean length time of the recordings was 59.77 ± 5.07 min, while the segments used for the analysis had a duration of 12.75 ± 0.72 min (mean ± SEM). No statistically significant differences were observed between the Sham- and not-lesioned animals for any of the characteristics analyzed in the present work, which will therefore be reported as a single group and referred to as control group.

### Discharge rate and statistical properties

**Figure 4:**
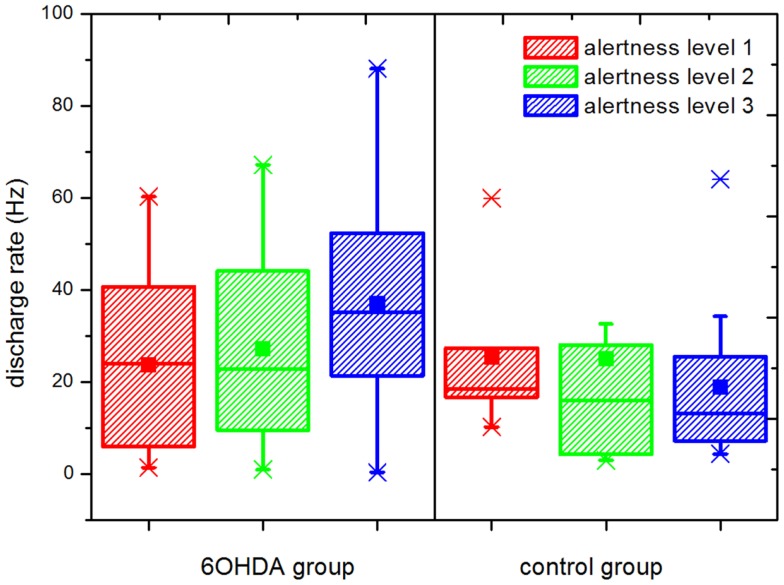
**Rate of discharge at different levels of alertness**. An increment in the discharge rate is observed in the parkinsonian neurons following increasing levels of alertness. A statistically significant difference was observed between the parkinsonian (*n* = 17) and control (*n* = 21) neurons at alertness level 3 (*p* ≤ 0.05), showing that this difference depends on the input received by the basal ganglia network. Upper and lower edges of the boxes represent percentile 75 (p75) and percentile 25 (p25) respectively, the inner line indicates the median, upper, and lower whiskers account for p75 + 1.5 (p75–p25) and p25–p1.5 (p75–p25), respectively. The mean is represented by the inner, solid box. Crosses indicate outliers.

Results for statistical properties and BTS-index of the time series are reported in Table 1. Statistical tests were run for comparisons between groups and significant differences are highlighted. The rate of discharge in the control group showed a decreasing trend as alertness increased, whereas in the parkinsonian group an increasing trend was observed (Figure 4). Under these conditions, while the control group presented a slightly higher discharge rate than the parkinsonian group (not significant) under the effect of anesthesia, the rate was significantly higher for the parkinsonian group in the fully alert state (alertness level 3, *p* ≤ 0.05).

**Table 1:** **Statistical properties of the time series analyzed for the different groups**.

	Parkinsonian group, alertness level 1 (*n* = 11)	Parkinsonian group, alertness level 2 (*n* = 17)	Parkinsonian group, alertness level 3 (*n* = 13)	Control group, alertness level 1 (*n* = 10)	Control group, alertness level 2 (*n* = 20)	Control group, alertness level 3 (*n* = 11)
Time series length (interspike intervals, ISI)	14.4 10E3 ± 4.5 10E3	18.0 10E3 ± 3.8 10E3	25.8 10E3 ± 6.9 10E3	19.4 E3 ± 4.1 10E3	16.7 10E3 ± 4.7 10E3	18.0 10E3 ± 5.2 10E3
Mean frequency (Hz)	21.88 ± 5.98	27.26 ± 5.25	37.02 ± 6.64*	25.37 ± 4.96	25.26 ± 8.09	18.89 ± 5.29*
Mean ISI (ms)	208.57 ± 82.23	181.67 ± 86.94	242.86 ± 175.01	55.10 ± 8.39**	120.03 ± 24.88**	98.17 ± 20.26
SD (ISI)	356.59 ± 134.52	282.13 ± 132.42	266.11 ± 184.88	55.21 ± 11.74***	169.02 ± 44.65***	116.97 ± 27.70
Skewness	3.09 ± 0.51	4.47 ± 1.10	3.14 ± 0.46	2.85 ± 0.50	2.43 ± 0.16	2.22 ± 0.20
Kurtosis	17.39 ± 6.90	55.86 ± 34.08	19.77 ± 6.16	26.47 ± 14.41	10.05 ± 1.40	8.32 ± 1.48
Coefficient of variation	1.53 ± 0.24	1.49 ± 0.19	1.13 ± 0.05	1.05 ± 0.17	1.16 ± 0.10	1.10 ± 0.14
Mode (ISI)	10.24 ± 5.00	13.18 ± 4.58	5.55 ± 1.51	22.41 ± 9.33	13.49 ± 4.26	14.96 ± 7.48
BTS-index (burst triggering spikes, see text)	2.06 ± 0.19	1.94 ± 0.13	1.76 ± 0.14	2.21 ± 0.19	2.08 ± 0.12	1.73 ± 0.21

Comparisons between groups were made and *p* ≤ 0.05 was considered significant. Significant differences are highlighted, *p = 0.04, **p = 0.02, and ***p = 0.02. ISI, interspike intervals. In row 5, SD refers to the standard deviation of the ISI distribution. Results are presented as mean ± SEM. The statistics shown were calculated over the whole length of the time series used for the analysis, which had duration of 12.75 ± 0.72 (mean ± SEM).

### Entropy

We analyzed the dependence of SE with the length of the time series (*n*) and with the embedding dimension for all the time series included in the study. The value of SE showed asymptotic behavior toward larger *n*. We found a stable value of SE for *n* ≥ 2500 for all time series. All the results presented here were obtained with *n* = 2500 ISI, and time series shorter than that were excluded. Regarding embedding dimension, SE was calculated for dimensions between 1 and 10 and relative consistency (meaning no changes in the relation between different time series) was found for dimensions between 3 and 5. This analysis was conducted using: first, all the available data points and second, *n* = 2500 data points for each time series and differences were not observed. All the results presented here were calculated considering an embedding dimension equal to 5.

**Figure 5:**
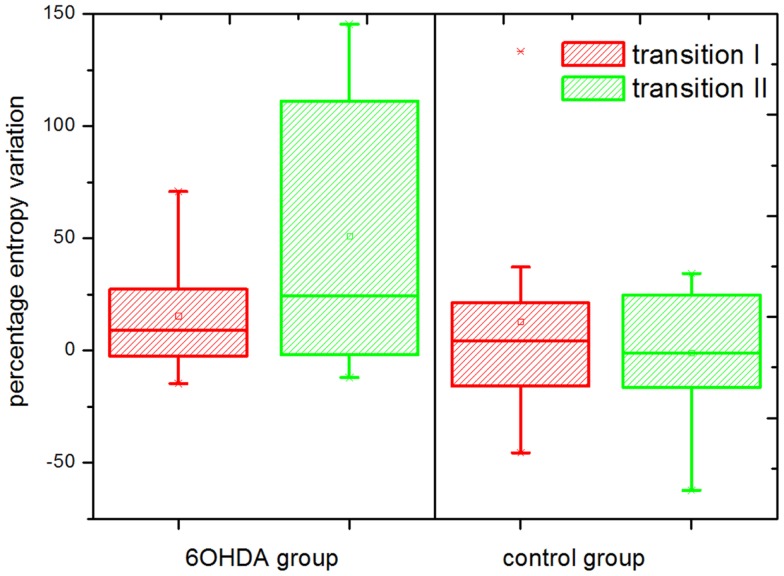
**Percentage entropy variation**. Transition I and II indicate the percentage variation in the level of sample entropy during the transition from alertness level 1–2, and 2–3, respectively. The entropy variation during transition II was significantly higher in the parkinsonian (6OHDA) than in the control group (*p* ≤ 0.05). A number of *n* = 2500 data points and an embedding dimension of five were used for the calculation of sample entropy. The boxes edges represent percentile 75 (p75) and percentile 25 (p25), respectively, the inner line indicates the median and whiskers account for p75 + 1.5 (p75–p25) and p25–p1.5 (p75–p25), respectively. The mean is represented by the inner, solid box. Crosses indicate outliers.

The results for the percentage entropy variation can be seen in Figure 5. With our selection of parameters, a significantly different behavior was observed in the parkinsonian model vs. control groups during the transition from alertness level 2–3 (i.e., from mild to full alertness: transition II). During transition II, an increase in the level of entropy was observed in the parkinsonian neurons, and the percentage of positive variation was higher than during the transition I in the same group (from alertness level 1–2). On the contrary, neurons of the control group showed a negligible variation of SE during transition II, even smaller than during transition I. The difference in the SE variation between control and parkinsonian neurons was statistically significant at a 95% confidence level, *p* ≤ 0.05. The strength of these results is enhanced by the fact that changes in single neurons across different levels of alertness were recorded, narrowing the effects of population variability.

## Discussion

Our results are in agreement with those found in the literature, where a decrease in the mode and an increase in the skewness, coefficient of variation, and kurtosis were found in alert parkinsonian rats (55). Regarding the frequency of discharge, some works have shown an increment in neurons from the 6OHDA-rat under anesthesia in comparison to controls, while others did not find statistical differences (56). We did not find statistically significant differences between the groups studied in the rate of discharge under the effect of anesthesia, but as the level of alertness increased the rate of discharge of the neurons in the 6OHDA group was increased as well, while a slight decreasing trend was observed in the control group. A significant difference was found in the fully alert state (alertness level 3) between parkinsonian and control neurons, with a higher discharge rate being observed in the 6OHDA group. The smooth changes observed in the rate of discharge of single cells following increasing levels of alertness highlight the importance of a careful quantification of the anesthesia depth in neurophysiological experiments.

Different methods are available for the calculation of entropy based on time series data (57, 58). We used the SE algorithm, which was shown in the literature to be a robust measure for short time series and to demand low computational cost. A positive relation between the level of alertness and the percentage SE variation in the discharge of single neurons in the parkinsonian rat GPi was documented. With a purely statistical approach, it can be shown that the maximum level of entropy of the output nodes of an arbitrarily connected network depends on the level of internal connectivity and the input to the network (59). Experimental evidence in favor of an excessive neuronal connectivity in the parkinsonian BG comes from studies, which showed an increment in the number of inter-neuronal connections in the parkinsonian Str (60). Although these studies haven’t been conducted in the GPi yet, it is possible that the number of inter-neuronal connections might be increased also in the GPi and other BG centers in the parkinsonian state. On the other hand, dopamine is known to act modulating the coupling between neurons (61). Therefore, it could also be expected for active mechanisms to be present in the healthy BG reducing the available connections in the alert state, which might offer an explanation for the negligible SE variation during the awakening process in the control group. In the parkinsonian group, our results show clearly that the increase of entropy is manifested during the transition toward the highest level of alertness. This reflects that in PD, the level of entropy in the discharge of single GPi neurons depends on the way the BG process incoming information. If the increased entropy variation observed is as well related to a pathological amount of inter-neuronal connectivity within the GPi is a question that remains to be elucidated in future works.

Summarizing, our experiments show that the entropy of GPi neurons *in vivo* varies dynamically depending on the input to the BG network. Therefore, we conclude that entropy needs to be interpreted as a dynamic, emergent property that characterizes the global state of the BG neuronal network, and should not be thought of as a static property of parkinsonian neurons themselves. Regarding the recently proposed entropy hypothesis of movement disorders, this dependence of the GPi neuronal entropy on the global input to the BG network in PD means that with increased BG input (e.g., at full alertness), the communication between the BG and the motor Th/cortex becomes progressively disrupted, which leads to a worsening of akinesia. Since our work was conducted with animals in relaxed, head-restrained condition and devoid of visual or auditory stimuli, it remains for future studies to analyze the relation between the level of entropy at the output centers of the BG, and specific sensory input or motor activity. Finally, the fact that in PD the entropy levels measured from BG neurons at full alertness is not directly comparable with measurements obtained under anesthesia is relevant to the implantation surgery of DBS electrodes, where the use of local vs. general anesthesia is still under discussion (62).

## Conflict of Interest Statement

The authors declare that the research was conducted in the absence of any commercial or financial relationships that could be construed as a potential conflict of interest.
